# Vitamin D Enhances Immune Effector Pathways of NK Cells Thus Providing a Mechanistic Explanation for the Increased Effectiveness of Therapeutic Monoclonal Antibodies

**DOI:** 10.3390/nu15163498

**Published:** 2023-08-08

**Authors:** Konstantinos Christofyllakis, Frank Neumann, Moritz Bewarder, Lorenz Thurner, Dominic Kaddu-Mulindwa, Igor Age Kos, Vadim Lesan, Joerg Thomas Bittenbring

**Affiliations:** Department of Internal Medicine 1, Oncology, Hematology, Clinical Immunology and Rheumatology, Saarland University Medical Center, 66421 Homburg, Germany; konstantinos.christofyllakis@uks.eu (K.C.); frank.neumann@uks.eu (F.N.); moritz.bewarder@uks.eu (M.B.); lorenz.thurner@uks.eu (L.T.); dominic.kaddu@uks.eu (D.K.-M.); igor.kos@uks.eu (I.A.K.); vadim.lesan@uks.eu (V.L.)

**Keywords:** vitamin D, lymphoma, rituximab, antibody-dependent cellular cytotoxicity, natural killer cells, interferon alpha, gene expression analysis, pathway analysis

## Abstract

Patients with diffuse large cell lymphoma who have an adequate vitamin D supply derive significantly more benefit from immuno-chemotherapy with rituximab than patients with vitamin D deficiency; this is especially true for female patients. We have already been able to show that vitamin D increases the antibody-dependent cytotoxicity (ADCC) of NK cells in a sex-dependent manner, but it is unclear how vitamin D makes NK cells more efficient. Methods: Healthy individuals with vitamin D deficiency were supplemented with vitamin D to sufficient levels. NK cells were isolated from blood samples before and after vitamin D saturation. For transcriptome analysis, we used the Affymetrix Gene-Chip 2.0™. Gene expression analysis as well as supervised and unsupervised pathway analysis were performed. Results: Among others the “NK cell-associated cytotoxicity pathway” increased after vitamin D substitution. Five IFN-α subtypes (2, 4, 6, 7 and 10) and IFN-κ were more highly expressed and are mainly responsible in these pathways. In contrast, the pathway “interferon-gamma response”, as well as other sets in cytokine production and chemotaxis showed a reduction. Toll-like receptor genes (TLR-8, TLR-7, TLR-2) were downregulated and, therefore, are responsible for the decline of these pathways. The same could be shown for the “ubiquitin-ligase” pathway. Conclusions: Increased expression of several IFN-α subtypes may explain the increased ADCC of NK cells in vitamin D-replenished and otherwise healthy subjects. Other regulators of interferon production and ADCC are compensatory upregulated in compensation, such as Toll-like receptors and those of the ubiquitin ligase, and normalize after vitamin D substitution.

## 1. Introduction

Vitamin D (cholecalciferol) is a secosteroid that plays a crucial role in maintaining bone health and regulating calcium levels in the body [1]. Cholecacliferol is activated by hydroxylation on position C25 by CYP27A1 in mitochondria or CYP2R1 in the endoplasmic reticulum and finally by CYP27B1 at position C1 alpha [2]. Alternative activation by CYP11A1 leads to a plethora of vitamin D variants which may cause many different modes of activation [2]. 

Therefore, it also has important immunomodulatory effects which are not yet fully understood [3]. Substitution of vitamin D affects the gene expression of several genes in human peripheral blood mononuclear cells [4]. Specifically, natural killer (NK) cells need vitamin D receptors for a proper development [5]. Therefore, it is no surprise that vitamin D is beneficial for NK cell function in the very elderly [6] or dialysis patients [7]. Furthermore, in patients with aggressive B cell lymphoma, vitamin D has helpful effects on the immune system antitumor response by activating macrophages [8]. In the context of modern cancer treatment, the question arises if the immunomodulatory effects of vitamin D can be harnessed to improve the antitumor effect of immunotherapy.

Therapeutic monoclonal antibodies (mAb) have had significant impact on lymphoma therapy in the last 25 years. The anti-CD20 mAb Rituximab is currently standard of care in all B cell malignancies [9], whereas the newer anti-CD20-mAb Obinutuzumab is used in indolent lymphoma and chronic lymphocytic leukemia [10,11]. 

Our group has been able to demonstrate, in a post-hoc analysis of a prospective phase III trial evaluating rituximab inpatients with diffuse large B-cell lymphoma in a randomized fashion, that patients who have adequate vitamin D serum levels derive significantly more benefit from the addition of rituximab to standard chemotherapy than patients with vitamin D deficiency [12], and, this is especially true for female patients. The main effector cell of therapeutic monoclonal antibodies are natural killer (NK) cells supported by macrophages, neutrophils and the complement system [13]. 

To further understand the above finding, healthy volunteers with vitamin D deficiency were supplemented with vitamin D up to predefined target serum levels. NK cells derived from these volunteers at various vitamin D serum levels were used to perform cytotoxicity assays against lymphoma cell lines in the presence of rituximab. We were able to show that vitamin D increases the antibody-dependent cytotoxicity (ADCC) of NK cells in a sex-dependent manner [14]. It still, however, remains unclear how vitamin D affects the NK cells to make them more efficient. In the present study, a transcriptome analysis from NK cells derived from those healthy volunteers was performed to identify the underlying mechanism behind increased NK-cell-mediated ADCC in the presence of rituximab after vitamin D supplementation.

## 2. Materials and Methods 

### 2.1. Treatment

Eight volunteers who had insufficient vitamin D levels were substituted orally with 20,000 IE capsules of 25-OH-vitamin D3 (Dekristol^TM^) (Mibe GmbH Arzneimittel, Brehna, Germany) to a mid-normal range. The dose was calculated using a modified version of the Van Groningen formula [15]. Depending on the body weight of the subject and the baseline 25-(OH)_2_-D3 vitamin status, volunteers took between 30 and 50 Dekristol^TM^ capsules (600,000–1,000,000 IE) over 6–10 days. Serum 25-OH Vitamin D was measured by chemoluminescence assay (LIASION^®^, DiaSorin, Saluggia, Italy) immediately prior to the NK cell assay, which was scheduled two weeks after supplementation.

The characteristics of the volunteers and the serum levels prior to and after supplementation are summarized in Table 1.

All participants gave written informed consent, and the Ethics Committee of the Saarland Medical Association (178/17) approved the study.

### 2.2. Isolation of NK Cells 

PBMCs were isolated by density gradient centrifugation from the 50 mL EDTA blood donation of the respective donor. Thereafter, NK cells were isolated from PBMCs by magnetic depletion of all non-NK cells using the CD56+/CD16+ human NK-Cell Isolation Kit (Miltenyi Biotech GmbH, Bergisch Gladbach, Germany) according to the manufacturer`s instructions. NK cells were isolated immediately before the ADCC assay without additional activation (e.g., by IL-2). The viability of the NK cells after isolation averaged 99% and the share of the CD16+ fraction was between 90% and 98%, as assessed by flow cytometry using the corresponding antibodies (Miltenyi). The yield of CD16+ NK cells was between 3 × 10^6^ cells and 1 × 10^7^ cells. A FACSCalibur (BD Biosciences, Heidelberg, Germany) was used for flow cytometry analysis. Starting with a forward scatter (FSC) versus sideward scatter scan (SSC) to gate the lymphocyte population and followed by analysis of the corresponding fluorescence using CellQuest software (BD Biosciences), 5000 or 10,000 lymphocytes were examined per run.

### 2.3. RNA Extraction

The isolated NK cells had been previously used in an ADCC assay on lymphoma cells. The excess NK cells were frozen at −80 °C, containing 5 × 10^6^ to 1 × 10^7^ NK cells. After centrifugation for the culture medium removal, RNA isolation was performed using the miRNeasy Mini Kit^TM^ (Qiagen, Velno, The Netherlands). A volume of 700 µL QIAzol Lysis Reagent^TM^ was added to the cells. Homogenization was performed with a QIAshredder Spin Column^TM^ (Qiagen). Then, 700 µL of the lysate was added to spin columns and centrifuged. Next, 140 µL chloroform was added to the tubes, mixed for 15 s and left at 22 °C for 2 min. A second centrifugation step at 12,000× *g* at 4 °C for 15 min followed. The aqueous phase was transferred to fresh tubes and we added 525 µL of 100% ethanol. This mixture was added to the RNeasy^TM^ Spin column in 2 mL tube and centrifugated at 8000× *g* for 15 s. This was repeated after addition of 500 µL RPE buffer^TM^. The spin column was then transferred to a new 2 mL collection tube and centrifuged at full speed for one minute to prevent carryover of flow-through into the RNA elution phase. Finally, the spin columns were placed into new 1.5 mL collection tubes, and 30 µL RNase-free water was pipetted onto each sample and then centrifuged at 8000× *g* for 1 min. 

### 2.4. Expression Analysis per Microarray

RNA expression analysis was performed with the GeneChip™ Human Gene 2.1 ST Array Plate (Thermofisher, Waltham, MA, USA). Microarray hybridization and sample preparation was carried out at “KFB—Center of Excellence for Fluorescent Bioanalytics” (Regensburg, Germany) with the GeneChip WT PLUS Reagent Kit Thermofisher, Waltham, MA, USA) as described in the manual. We generated double-stranded cDNA from 200 ng of total RNA. Subsequently, 12 µg cRNA was synthesized, purified and reverse-transcribed into sense-strand (ss) cDNA, while incorporating unnatural dUTP residues. Fragmentation of purified ss cDNA was performed using a combination of uracil DNA glycosylase (UDG) and apurinic/apyrimidinic endonuclease 1 (APE 1). Finally, terminal labeling with biotin was performed, and 3.8 µg of each ss cDNA sample was used for hybridization. A GeneTitan system, controlled by the GeneChip Command Console software v4.2 (Thermofisher, Waltham, MA, USA), was used for hybridization, washing, staining and scanning. Signal strengths were normalized and log_2_-transformed. Quality controls according to the manufacturer’s instructions were performed prior to and after normalization without identification of any outliers.

### 2.5. Validation

Selected genes were validated using quantitative Real-Time Polymerase Chain Reaction (qRT-PCR). Genes with significant, divergent across samples expression and available primers were selected (allograft inflammatory factor 1, AIF1 and C-type lectin domain family 7, member A, CLEC7A). One housekeeping gene was chosen to act as a control: ubiquitin-conjugating enzyme E2D 2 (UBE2D2). Primers for the above mentioned genes were synthesized (Sigma-Aldrich, Darmstadt, Germany), followed by reverse transcription, cDNA synthesis and RT-PCR.

### 2.6. Statistical Analysis 

At a first step, differentially expressed genes were analyzed. We initially performed a Kolmogorov–Smirnov test to confirm the normal distribution of the results using SPSS (IBM SPSS statistics, version 23). For the comparison of gene expression after vitamin D supplementation, the “R” software (R Foundation for Statistical Computing, version 3.6.3) with the “limma” package (Bioconductor, version 3.42.2) through the RobiNA graphical interface (version 1.24) was used with the default settings. Bonferroni correction for multiple correction was used and significance was defined at *p* < 0.01. For the subgroup analysis according to sex and vitamin D, we performed two-sided analysis of variance (ANOVA) with the Transcriptome Analysis Console (TAC) Software (Thermofisher Scientific, 4.0.2 Release) and its integrated “limma” package (Bioconductor, version 3.42.2).

We used the “Gene Set Enrichment Analysis” (GSEA) [16] software and the online tool GeneTrail to detect molecular pathways which were affected by vitamin D supplementation.

The default metric Signal2Noise (the difference in means scaled by the standard deviation) and the weighted (*p* = 2) enrichment statistic were selected for GSEA. The cut off for *p* was set at <0.05. 

First, the “NK cell-associated cytotoxicity pathway” from the Kyoto Encyclopedia of Genes and Genomes (KEGG) database was examined using only 7705 genes known to be involved in the NK-cell-mediated immune response, to increase the sensitivity. Second, the entire gene dataset was used in an supervised analysis in pathways involved in the immune system in general (*n* = 89 pathways), from databases such as “Biocarta”, “KEGG”, “Gene ontology (GO)”, and “Reactome” databases, all accessed through the Molecular Signature database. Both analyses were performed with the GSEA software, version 4.0.3, Broad Institute. When several pathways were enriched, we performed a leading-edge analysis to see which genes contributed the most to the observed pathway changes.

Finally, Gene Trail was used in an unsupervised manner to screen for pathways in the “Hallmark”, “BioCarta”, “Biological Process”, “Cellular Component”, “Molecular Function”, “KEGG”, “Reactome” and “WikiPathways” online databases, without any preselection of neither genes nor pathway category. Again, a weighted GSEA was used. For this unsupervised analysis, we decided to adjust the *p*-value for significance to <0.01 as this was a non-hypothesis driven test. Adjustment for multiple testing was conducted according to Benjamini–Hochberg.

## 3. Results

Supplementation was performed successfully, leading to an increase in vitamin D serum levels from 10.5 ng/mL to 65.6 ng/mL (Table 1).

### 3.1. Gene Expression Changes in Vitamin-D-Supplemented Volunteers

In total, 505 transcripts changed their expression levels after vitamin D administration with a cutoff *p* < 0.01. Only 256 had an annotation to a specific gene locus (coding and non-coding). Up- and downregulated transcripts are listed in Table 2. Among others, the *IFNL3* gene, encoding IFN-λ3 (or IL28B) and *IL17RE* (interleukin 17 receptor E), as well as *IL2RB* (beta subunit of the interleukin receptor 2) were upregulated after administration of 25-(OH)2D3.

**Figure 1:**
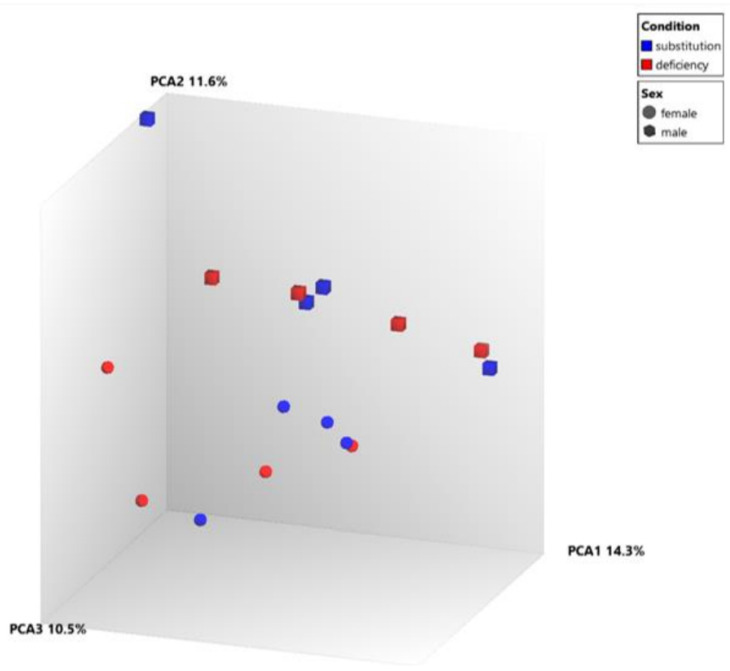
Principal component analysis—1 Red indicates samples before and blue after vitamin D supplementation. Cubes indicate males and spheres indicate females. The individual samples do not seem to form clusters based on vitamin D levels. Thus, no clearly changed expression pattern could be observed which clearly is attributable to supplementation. However, a clear spatial separation for sex is noted (males concentrate in the upper part of the diagram, females in the lower part).

**Figure 2:**
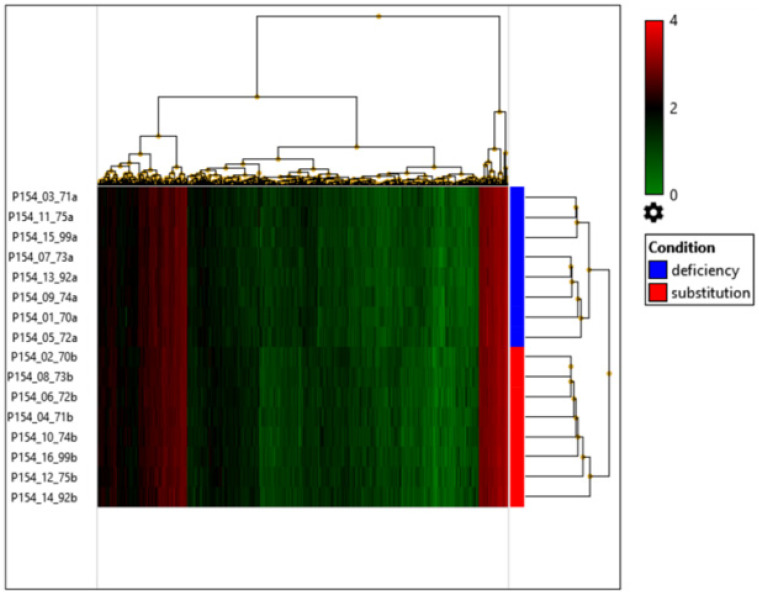
Hierarchical clustering based on the expression profile of transcripts that are up- or downregulated based on *p* < 0.05 across the two different vitamin D levels. The color scale represents log_2_-transformed expression of the single gene samples. Brighter green represents lower expression and brighter red represents higher expression. No gene clusters which clearly separate the two states could be identified.

In the principal component analysis, in the samples we did not find specific gene clusters based on vitamin D levels (Figure 1) and the heatmap displays that vitamin D substitution had only a small effect on the NK cell transcriptome as such (Figure 2).

After Bonferroni correction for multiple testing, no gene was significantly differentially expressed in the analysis. Up- and downregulated transcripts are listed in Table 2. Table 3 shows differentially expressed genes associated with the immune system or vitamin D signal transduction according to the GO database.

Sex-specific differences in gene expression are shown in Table S1. In males, among others, the *IFNL3* gene is significantly upregulated, while the *IFNG* gene is downregulated. In females, amidst other changes, the toll-like receptor genes *TLR5* and *TLR8* are downregulated.

### 3.2. Pathway Analysis

For GSEA pathway analysis, a ranked gene list was created according to the signal-to-noise metric. The 50 most up- or downregulated transcripts on the list are presented in a heatmap in Figure S1.

**Figure 3:**
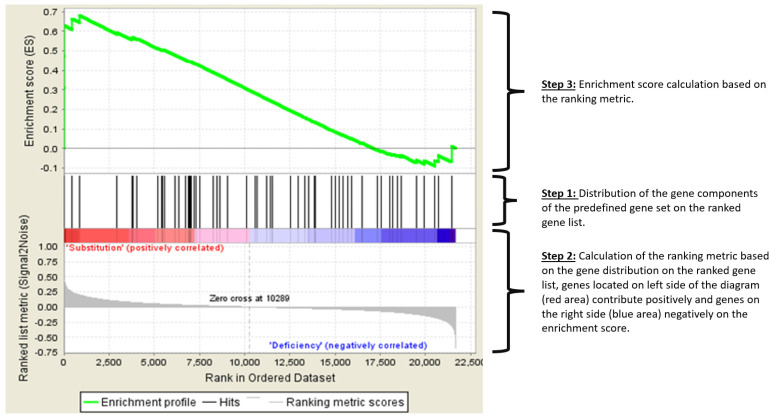
Enrichment Plot: “NK cell cytotoxicity” pathway. GSEA firstly creates a ranked list of differentially expressed genes across the dataset ranging from the highest change towards one phenotype (e.g., after substitution) to the highest change towards the other phenotype. Then, a predefined set of genes with a common biological function, e.g., components of one molecular “pathway” is compared against the ranked gene list. The GSEA then determines whether the genes belonging to this pathway are randomly distributed in the ranked list, or whether they are found at the top or bottom of the list. If a set of genes has a distribution associated with a particular phenotype (top or bottom of the list), the extent of the correlation is measured with an enrichment score (ES). The upper part of the figure shows the running ES for the gene set as the analysis walks down the ranked list. A positive ES (the green line is mostly above 0.0) means overexpression in the particular phenotype, in this case after vitamin D substitution. The middle part of the figure shows the placement of the individual genes of a certain set in the ranking of all genes. Each gene is represented by a line. The bottom part of the plot shows the value of the ranking metric, indicating the correlation of a gene to either one of the two phenotypes “substitution” or “deficiency”. It is positive (red) when the gene shows higher expression in the phenotype “substitution” and negative (blue) when expression is higher in the phenotype “deficiency”.

Importantly, the “NK cell-associated cytotoxicity pathway” from the KEGG database was shown to be upregulated. A gene set enrichment plot is shown in Figure 3 and the main genes responsible for the amplification of the pathway are shown in Table 4, and consist mostly of interferon alpha genes.

In the following part, the entire gene expression profiles by microarray were analyzed. With A cutoff *p* value < 0.05 we identified two pathways to be upregulated: the pathway “Regulation of type I interferon-mediated signaling” and the pathway “Response to type I interferon”. A leading-edge analysis identified the genes which contributed the most. As in the NK cytotoxicity pathway, five interferon alpha genes, *IFN2,*-*4,*-*6,*-*7* and -*10*, had the highest expression change in these pathways.

**Figure 4:**
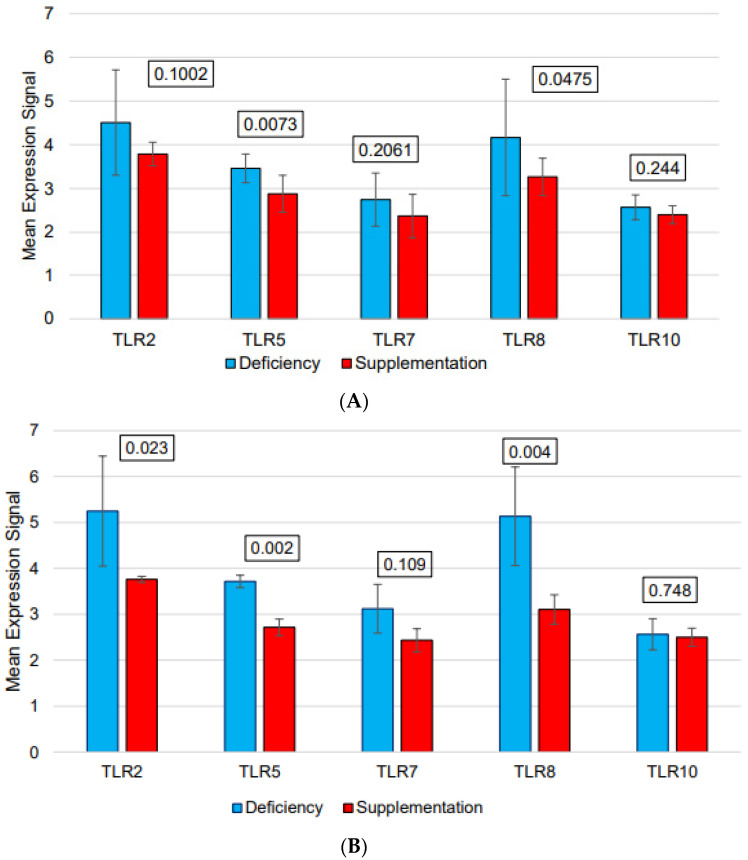
Selected TLR gene expression before (blue columns) and after (red columns) vitamin D supplementation (**A**): across all subjects. (**B**): only in females. Standard deviation is shown as error bar. The unadjusted *p* values according to two-way ANOVA are given above each pair of columns. Across all subjects, of the five TLR genes, TLR5 and TLR8 were downregulated (*p*-value < 0.05) after vitamin D supplementation.

We found eleven downregulated pathways related to immune functions (Table 5). Additionally, we performed a leading-edge analysis to see which genes contributed the most to this plethora of pathway changes. Toll-like receptors 2, 7 and 8 were involved in five out of the eleven altered pathways (Figure 4).

Additionally, in an unsupervised analysis, not restricted to the immune-related pathway, we found a significant downregulation of the E3 ubiquitin ligase pathway. 

## 4. Discussion

Vitamin D substitution did not alter individual gene expression in NK cells after statistical correction for multiple testing. However, additional bioinformatical methods were able to demonstrate the transcriptomic background of the effect of vitamin D on NK cell activity. 

GSEA determines whether the distribution of amplified genes are closely correlated with a particular pathway. The extent of correlation is measured by the enrichment score. GSEA can give additional insights in finely tuned regulation of gene expression changes by endogenous hormones or pro-hormones, such as vitamin D, which had a very long presence in the animal phylogeny to exert its effects rather subtly.

We could show that the “NK cell-associated cytotoxicity pathway” from the KEGG database among other Interferon-related pathways was significantly upregulated after vitamin D substitution. This is mainly caused by upregulated IFN-α genes (*IFN2*, -*4*, -*6*, -*7*, and -*10*) and this finding is in line with the expectation we had from our clinical [12] and translational [14] results of vitamin D activity and NK-cell-mediated ADCC. Also, *IFNL3* was shown to be upregulated. We, therefore, propose that increased interferon production plays the most important role in vitamin D enhancement of NK cell activity. Other cytokines (IL-2 and IL-17) may also be involved, since their receptor genes were shown to be upregulated. IL-2 is a well-established NK cell activator [17], and IL-17 increases circulating NK cells, perforin and granzyme expression, and finally cytolytic function [18].

The downregulation of the TLRs (*TLR2*, *TLR5*, *TLR7* and *TLR8*) in the vitamin-D-deficient state may seem at first paradox, as they are involved in the IFN-α response, due to exogenous signals such as pathogen-associated molecular patterns. This must, however, be considered in the context of the observed downregulation of the ubiquitin ligase pathway. 

The ubiquitin ligase system is a known inhibitor of NK cell activity [19]. This is further supported by data demonstrating that inhibition of ubiquitin ligases in a mouse model [20] leads to remission of tumors by NK cell lysis. Furthermore, viral evasion of NK cell activity has been shown to be induced by the production of E3 ubiquitin ligases in human herpesvirus 8 [21]. Importantly, this ubiquitin ligase-dependent immune suppression is, at least in part, induced by regulation of the TLR/IFN axis. Ubiquitin ligases have been shown to inhibit TLR signaling through nuclear factor (NF)-kB [22] and their knockdown increases proinflammatory cytokines via the TLR/IL-1R/TRAF6 pathway [23]. Production of IFN-α upon TLR stimulation is known to depend on the formation of a complex consisting of MyD88, IRF7 and TRAF6, as well as TRAF6-dependent ubiquitination [24]. Most importantly in the context of our results, ubiquitin ligases inhibit type I IFN secretion (including IFN-α) through IRF3 and IRF7 ubiquitination [25].

Therefore, one coherent pattern emerges: the ubiquitin ligase downregulates type I interferon synthesis and is an inhibitor of NK cell activity, which is itself reduced by vitamin D, so substitution of vitamin D leads to increased IFN-α production. This would explain the increased NK cell activity observed after supplementation. TLR overexpression in the vitamin D deficiency state may be compensatory for the suppression of the type I interferon production pathway, which is triggered by ubiquitin ligase activity. We hypothesize a compensatory upregulation of TLRs, as in the vitamin-D-deficient state the NK cells are less likely to be successful in their task and, therefore, increase their activating receptors for exogenous stimuli.

The downregulation of *IFNG* is not necessarily inconsistent with the observed effect of increased NK cell ADCC against lymphoma. IFN-γ leads to increased expression of T-cell inhibitory ligands like PD-L1 in tumors [26]. Additionally, it causes overexpression of MHC-I molecules on target cells, which are inhibitory ligands for NK cells. However there is some experimental data that leukemia and lymphoma cell lines may be protected from NK cell activity in the presence of IFN-γ [26]. Increased NK cell activity, therefore, may be, in part, possible when IFN-γ is downregulated.

This study provides an explanation on the basis of gene expression data for the observation of increased NK-cell-mediated ADCC in the presence of the mAb rituximab. Newer therapeutic agents in lymphoma therapy such as antibody drug conjugates like polatuzumab–-vedotin [27] would not be predicted to be affected by vitamin D levels, as they do not rely solely on host immune mechanisms, but rather on the toxic component. Whether vitamin D influences the effect of bispecific antibodies like glofitamab and epcoritamab, which rely on T-cell-mediated direct cytotoxicity, remains to be discovered.

Considering the study design, our approach has the advantage of isolating the effect of vitamin D supplementation on NK cells through serial testing of the same individuals. Thus, confounders are limited to a minimum. The present study used NK cells from the same probands, for which our group has demonstrated increased NK-cell-associated ADCC in the presence of rituximab against lymphoma cells after vitamin D supplementation to target levels [14]. Thus, a mechanistic explanation for the increased NK cell activity is provided. However, the NK cells in this experiment have had no direct contact with target cells. It is possible that a transcriptome analysis of NK cells after they had interacted with lymphoma cells would demonstrate a different picture. Future experiments based on, for example, single cell RNA-Seq could answer this question.

## 5. Conclusions

We conclude that vitamin-D-deficient NK cells are less likely to produce IFN-α and are therefore less likely to kill a rituximab-coated target cell. Several pathways are upregulated to compensate but are not sufficient to restore NK cell activity. After the vitamin D is replenished, the NK cells restore IFN-α secretion and can more successfully exert ADCC. Increased expression of IL2 and IL17 receptors additionally supports this process. The analysis presented adds some information on how therapeutic monoclonal antibodies work in lymphoma treatment and gives additional hints how to improve and fully exploit the potential of monoclonal antibody therapy in malignancies.

## Figures and Tables

**Table 1:** Age distribution, pre- and post-supplementation vitamin D levels of the healthy participants.

	Sex	Age (Years)	Vitamin D Levels in Deficient Status (ng/mL)	Vitamin D Levels After Supplementation (ng/mL)
	male	78	5.9	64.3
	female	71	23	68.2
	male	57	15.7	62.6
	female	78	4.6	68.8
	male	79	6.1	72.8
	female	79	9.7	68.5
	male	86	10.3	58.2
	female	42	8.8	61.5
**Mean**		71.3	10.5	65.6
**Standard deviation**		14.6	6.1	4.8
**Median**		78	9.25	66.25
**Range**		42–86	4.6–23	58.2–72.8

**Table 2:** Up- and downregulated genes in NK cell isolates after vitamin D supplementation.

Upregulated Genes			Downregulated Genes		
*CDH19*	*MAP4K1*	*THOP1*	*ADORA2B*	*KIF15*	*UGT2B17*
*CELSR3-AS1*	*MED24*	*TOX2*	*AFF4*	*KIF1B*	*UPRT*
*CHST15*	*MIR1244-1*	*TP53I13*	*ANAPC4*	*KRTAP4-2*	*VWC2L-IT1*
*COQ3*	*MIR548T*	*TRAV8-4*	*ANP32B*	*LRRC1*	*ZEB2*
*CRELD2*	*MIR570*	*TRIM51HP*	*ANXA8L1*	*LRRFIP2*	*ZNF709*
*CRSP8P*	*MT1B*	*TTTY13*	*ASIP*	*MAF*	*ZNF839*
*CTDNEP1*	*MTRNR2L5*	*UCHL1*	*ATP11C*	*MBTD1*	
*CUEDC2*	*MYBL2*	*UPK3B*	*BBS5*	*METTL14*	
*CYP21A1P*	*MYO7A*	*UROS*	*BLOC1S6*	*MGC16025*	
*DDX39A*	*NAV2-AS5*	*USH2A*	*C15orf57*	*MIEF1*	
*DENND6B*	*NRG4*	*VAMP2*	*C16orf46*	*MIR1-1*	
*DMRTA1*	*OR52W1*	*VIP*	*CAMTA1*	*MIR140*	
*DRD2*	*P4HA2*	*WBSCR16*	*CCDC81*	*MIR3175*	
*EEF1A2*	*PCBP4*	*YKT6*	*CCR10*	*MIR330*	
*EIF5AL1*	*PERP*	*ZNF324*	*CCSAP*	*MIR4496*	
*ELFN1*	*PI4KA*	*ZNF692*	*CCT2*	*MIR933*	
*EPB41L1*	*PI4KAP1*	*ZNF728*	*CDC14C*	*NFATC4*	
*EXOC6B*	*PKD2L1*	*ZNF733P*	*CDH13*	*NKAIN2*	
*F9*	*PLXDC1*	*ZNF788*	*CDKL5*	*OPCML*	
*FAM189B*	*POLR2L*		*CEP57L1*	*OR6C70*	
*FAM217A*	*PPM1M*		*CHORDC1*	*PADI4*	
*FAM219A*	*PPP1R15B*		*CLIP4*	*PGRMC2*	
*FAM25C*	*PRAMEF2*		*CMSS1*	*PKN2*	
*FAM53A*	*PRPF31*		*CTRB1*	*PLA2G4A*	
*FBL*	*PRR23D2*		*CYTIP*	*PLEKHH2*	
*FBLN7*	*PRRT3*		*DEFB124*	*PRKCH*	
*FBXL6*	*PTPRCAP*		*DENND5B-AS1*	*PRR3*	
*FN1*	*PXMP2*		*DHX15*	*PSMC6*	
*GPR146*	*RARRES3*		*DICER1*	*PTGER4*	
*HIST1H4F*	*RASA4B*		*DISP2*	*RAD51AP2*	
*HLA-C*	*RASL10B*		*DNAJA4*	*RBM44*	
*HPSE2*	*RETN*		*DOCK5*	*RGPD1*	
*HULC*	*RHBDF1*		*DSCAM-IT1*	*RHOB*	
*IFITM5*	*RNU5D-1*		*EFCAB10*	*RIOK3*	
*IFNL3*	*RPH3AL*		*EHHADH*	*RPH3A*	
*IL17RE*	*RPL21P28*		*EMC2*	*RSPH10B*	
*IL2RB*	*RPL23AP87*		*EXOG*	*SAYSD1*	
*INO80E*	*RPS27*		*FAM208B*	*SCGB2B3P*	
*ITGAM*	*SEC11A*		*FBXW9*	*SCUBE1*	
*ITGAX*	*SH3GLB2*		*FEM1C*	*SELK*	
*IVL*	*SLPI*		*FETUB*	*SENP2*	
*KDM8*	*SMARCB1*		*FGD5P1*	*SENP5*	
*KIF25*	*SNAR-I*		*GABPB1-AS1*	*SERBP1*	
*KRT17*	*SNORA35*		*GACAT2*	*SLMAP*	
*KRTAP22-2*	*SNORD116-14*		*GCOM1*	*SMCHD1*	
*KRTAP5-1*	*SNORD20*		*GPATCH2L*	*SMNDC1*	
*LIN7A*	*SNORD32B*		*GS1-279B7.1*	*SNORD114-3*	
*LINC00619*	*SOX13*		*HACD1*	*SPINT1*	
*LINC00881*	*SPATA20*		*HOXD12*	*SRPK1*	
*LINC01144*	*SPINK1*		*HYAL2*	*TAOK1*	
*LINC01456*	*SSH3*		*IMMP1L*	*TPI1P3*	
*LINC01624*	*STARD4-AS1*		*IP6K2*	*TRBV5-4*	
*LRRC42*	*STARD9*		*IPO11*	*TSFM*	
*LRRC74A*	*TAAR3*		*KCNE1*	*TTC39B*	

**Table 3:** Genes associated with the immune function or vitamin D signaling according to the GO database. Decreased expression −, increased expression + fold change *p* < 0.01.

*Gene* *Symbol*	*Gene Name*	*Fold Change*	*p-Value*
*TRAV8–4*	T cell receptor alpha variable 8–4	−1.54	0.00761
*MAP4K1*	mitogen-activated protein kinase kinase kinase kinase 1	−1.34	0.00388
*IFNL3*	interferon, lambda 3	−1.30	0.00671
*IL17RE*	interleukin 17 receptor E	−1.24	0.00443
*POLR2L*	polymerase (RNA) II (DNA directed) polypeptide L, 7.6kDa	−1.23	0.00904
*VIP*	vasoactive intestinal peptide	−1.22	0.00146
*C1orf147*	chromosome 1 open reading frame 147	−1.21	0.00525
*IFITM5*	interferon induced transmembrane protein 5	−1.19	0.00350
*SLPI*	secretory leukocyte peptidase inhibitor	−1.19	0.00999
*DRD2*	dopamine receptor D2	−1.18	0.00661
*ITGAM*	integrin, alpha M (complement component 3 receptor 3 subunit)	−1.16	0.00232
*IL2RB*	interleukin 2 receptor, beta	−1.13	0.00802
*VAMP2*	vesicle associated membrane protein 2	−1.10	0.00815
*MED24*	mediator complex subunit 24	−1.09	0.00743
*NRG4*	neuregulin 4	−1.09	0.00871
*HLA-C*	major histocompatibility complex, class I, C	−1.08	0.00426
*CCR10*	chemokine (C-C motif) receptor 10	1.12	0.00455
*PSMC6*	proteasome 26S subunit, ATPase 6	1.13	0.00591
*PTGER4*	prostaglandin E receptor 4 (subtype EP4)	1.16	0.00388
*SRPK1*	SRSF protein kinase 1	1.18	0.00589
*NFATC4*	nuclear factor of activated T-cells, cytoplasmic, calcineurin-dependent 4	1.19	0.00520
*BLOC1S6*	biogenesis of lysosomal organelles complex-1, subunit 6, pallidin	1.22	0.00058
*DEFB124*	defensin, beta 124	1.23	0.00058
*TRBV5-4*	T cell receptor beta variable 5-4	1.30	0.00337
*PADI4*	peptidyl arginine deiminase, type IV	1.41	0.00820
*PLA2G4A*	phospholipase A2, group IVA (cytosolic, calcium-dependent)	1.67	0.00099

**Table 4:** Genes contributing to the enrichment score of the “Natural Killer Cell mediated cytotoxicity” pathway.

*Gene Symbol*	*Gene Name*
*IFNA10*	interferon, alpha 10
*IFNA6*	interferon, alpha 6
*IFNA4*	interferon, alpha 4
*IFNA2*	interferon, alpha 2
*PPP3R2*	protein phosphatase 3 (formerly 2B), regulatory subunit B, 19kDa, beta isoform (calcineurin B, type II)
*RAC3*	ras-related C3 botulinum toxin substrate 3 (rho family, small GTP binding protein Rac3)
*RAET1L*	retinoic acid early transcript 1L
*IFNA7*	interferon, alpha 7
*NCR2*	natural cytotoxicity triggering receptor 2
*IFNA13*	interferon, alpha 13
*SHC2*	SHC (Src homology 2 domain containing) transforming protein 2

Abbreviation: ES: Enrichment Score.

**Table 5:** Downregulated pathways after vitamin D substitution.

*Name of the Pathway*	*ES*	*NES*	*p-Value*	*FDR* *q-Value*
Biocarta—FCεRI	0.90	1.88	<0.001	<0.001
GO—Activation of innate immune response	0.71	1.87	<0.001	<0.001
Hallmark—Complement	0.70	1.84	<0.001	<0.001
GO—Interferon Gamma response	0.68	1.78	<0.001	<0.001
GO—Regulation of interferon beta production	0.80	1.71	<0.001	0.002
GO—Positive regulation of interferon beta production	0.86	1.70	<0.001	0.002
GO—Leukocyte chemotaxis	0.67	1.64	<0.001	0.013
GO—Positive regulation of interferon alpha production	0.89	1.59	0.002	0.032
Hallmark—IL6/JAK/STAT3 signaling	0.66	1.58	<0.001	0.032
GO—Activation of immune response	0.57	1.57	<0.001	0.032
GO—Response to vitamin D	0.77	1.57	0.012	0.031

Abbreviations: ES: Enrichment Score, NES: Normalized Enrichment Score, FDR: false discovery rate, GO: Gene Ontology.

## Data Availability

The data that support the findings of this study are deposited at the publicly available Gene Expression Omnibus database, reference number [GSE239308]. https://www.ncbi.nlm.nih.gov/geo/query/acc.cgi?acc=GSE239308. Raw data can be downloaded from 26 July 2024.

## Supplementary Materials

The following supporting information can be downloaded at: https://www.mdpi.com/article/10.3390/nu15163498/s1, Figure S1: Heat map of the 50 most significant transcripts for each phenotype as per GSEA ranked list; Table S1: Differentially expressed genes according to sex for a *p* value <0.01 and increase/decrease in fold change by at least 1.5.

## Abbreviations

ADCC	Antibody-dependent cellular cytotoxicity
ANOVA	Analysis of variance
FcyIIIA	low affinity IgG receptor
FcεR1	High affinity IgE receptor
GO	Gene ontology
GSEA	Gene set enrichment analysis
HER2	Human epidermal growth factor receptor 2
IFN	Interferon
KEGG	Kyoto encyclopedia of genes and genomes
mAb	Monoclonal antibody
NK	Natural killer
PBS	phosphate-buffered saline
PBMC	peripheral blood mononucleated cell
TLR	toll-like receptor
qRT-PCR	quantitative real time polymerase chain reaction
