# Supplementary material for: Vitamin D Enhances Immune Effector Pathways of NK Cells Thus Providing a Mechanistic Explanation for the Increased Effectiveness of Therapeutic Monoclonal Antibodies
Source: Nutrients. 2023 Aug 8;15(16):3498. doi: 10.3390/nu15163498 (PMC10457932; doi:10.3390/nu15163498)
Supplement: Supplementary file 1 [file nutrients-15-03498-s001.zip › nutrients-2481492-supplementary.pdf]

nutrients

Supplementary Materials

Vitamin D Enhances Immune Effector Pathways of NK Cells thus Providing a Mechanistic Explanation for the Increased Effectiveness of Therapeutic Monoclonal Antibodies

Konstantinos Christofyllakis [1], Frank Neumann [1], Moritz Bewarder [1], Lorenz Thurner [1], Dominic Kaddu-Mulindwa [1], Igor Age Kos [1], Vadim Lesan [1] and Joerg Thomas Bittenbring [1,*]

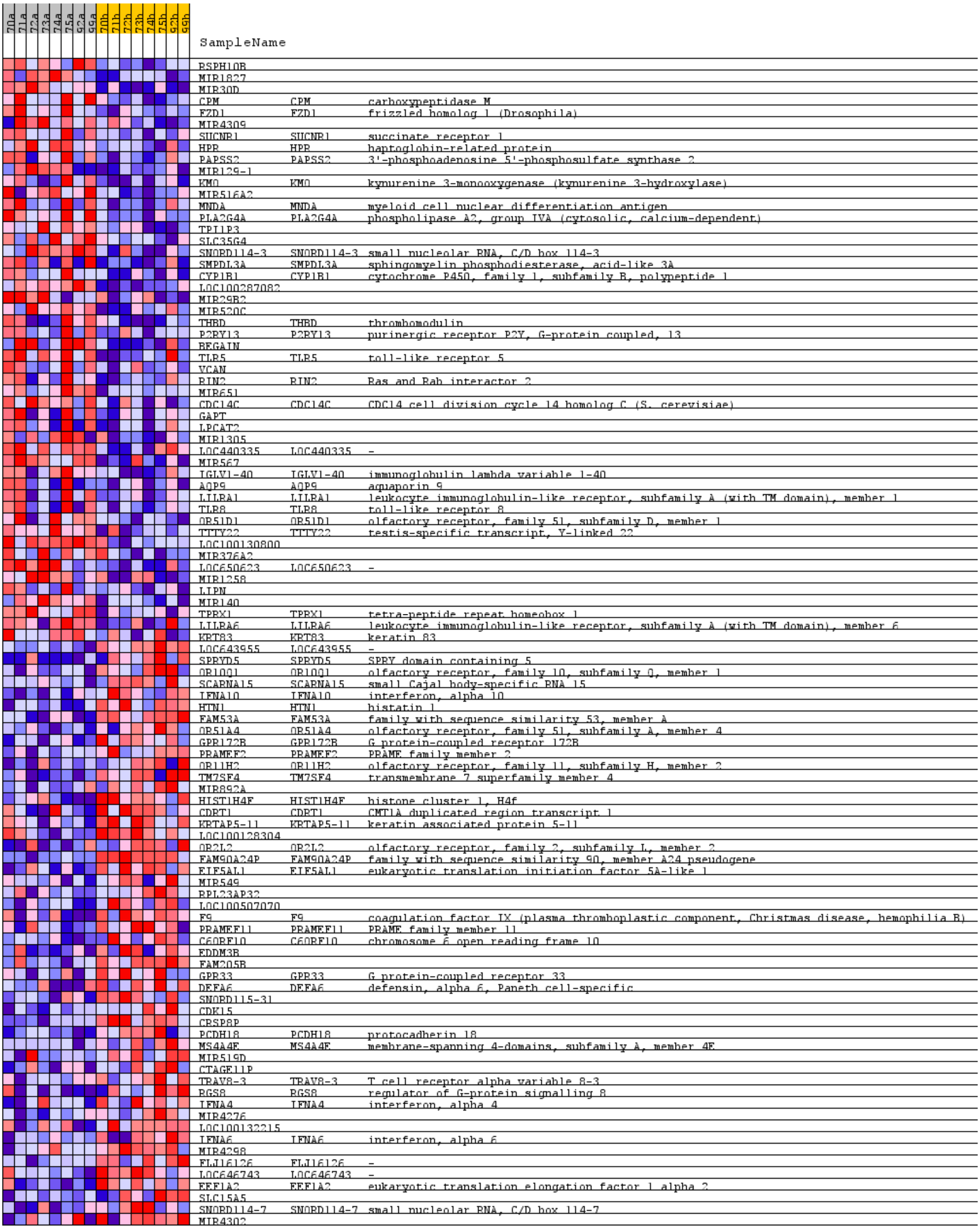

Figure S1. Heat map of the 50 most significant transcripts for each phenotype as per GSEA ranked list. The heatmap demonstrates the correlation between the ranked genes and the phenotypes (columns on the left: deficiency, columns on the right: substitution). The expression values are represented in a color scale, where the range of colors (red, pink, light blue, dark blue) shows the range of expression values (high, moderate, low, lowest). The metric used was the default Signal2Noise metric. Only the top 50 downregulated and top 50 upregulated transcripts are shown.

Table S1. Differentially expressed genes according to sex for a p value <0.01 and increase/decrease in fold change by at least 1.5.

Females		Males	
**Gene**	**Fold change**	**Gene**	**Fold change**
TRIM51	-2.44	*EIF5AL1*	-2.28
MIR3180-5; MIR3180-4	-2.06	*MIR548T*	-2.27
ZNF733P	-1.93	*MIR4476*	-2.17
TRBV7-6	**-1.93**	*GGT1; SNRPD3*	-2.16
WLS	-1.88	*ARHGAP39*	-1.82
KRTAP5-11	-1.87	*FGF12-AS3*	-1.81
SAMD5	-1.77	*TTTY14*	-1.71
MIR4330	-1.68	*MIR4279*	-1.68
NBPF13P	-1.66	*C9orf139*	-1.68
RGS8	-1.63	*SLC52A1*	-1.64
SYDE2	-1.61	*SERPINA12*	-1.64
IGKV5-2	**-1.6**	*TUBA3D*	-1.62
SNORD116-20; SNORD116@	-1.59	*MIR125A*	-1.61
MIR187	-1.57	*SEMA6A*	-1.59
ZNF649-AS1	-1.56	*LRRC3*	-1.59
OR5D14	-1.53	*EFCAB12*	-1.59
HULC	-1.52	*SNRPN*	-1.55
PART1	-1.51	***IFNL3***	**-1.54**
NPS	-1.5	*BIRC7*	-1.52
PTPRO	1.51	*PLA2G4E-AS1*	-1.51
PAK1	**1.56**	*USP51*	1.51
LINC00853	1.56	*C5orf60*	1.54
L1TD1	1.57	*ASAP1-IT2*	1.64
KCNE1	1.58	*TPRX1*	1.66
CEP295NL	1.6	*MGAM2*	1.83
ARHGAP5	1.6	*ZSCAN5CP*	1.9
CNP	1.62	*MIR1827*	1.92
SLC31A2	1.63	***IFNG***	**2.12**
SIGLEC5	1.64		
KDM1B	1.64		
USP12-AS2	1.67		
GCA	1.69		
MIR370	1.7		
DUSP3	**1.7**		
WFDC10B	1.72		
SLC24A4	1.73		
SLC37A2	1.77		
CD99P1	1.79		
FGD6	1.82		
RPL23AP7	1.83		
TLR5	**1.89**		
CPM	1.89		
MIR4309	1.91		
C9orf72	1.93		
MS4A6A	1.97		
FZD1	**2.09**		
SMPDL3A	2.27		

IGSF6	2.41
CFP	**2.68**
PAPSS2	2.89
GAPT	**3.24**
MNDA	3.74
TLR8	**3.77**
RNU6-53P	4.24

Genes involved in the immune response are highlighted. A negative fold change represents an upregulation, whereas a positive fold change represents downregulation after vitamin D supplementation.
